# Alcohol and the Hospitals

**Published:** 1901-07-20

**Authors:** J. J. Ridge

**Affiliations:** Hon. Secretary, British Medical Temperance Association. Carlton House, Enfield


### Editor's Letter-Box.

[Our Correspondents are reminded that prolixity is a great bar to publication, and that brevity of style and conciseness of statement greatly facilitate early insertion.]

### ALCOHOL AND THE HOSPITALS.

To the Editor of *The Hospital.*

Sir,—My reply to your anonymous correspondent need not be as long as his impeachment. It is not usual in journalistic circles to attribute an unsigned article to a particular individual, and to name that person again and again, mingling abuse with ridicule; I can well understand that he objects to sign his own name to his angry and discourteous remarks.

But having more respect for your readers than one can have for such an opponent, I look to you to allow me to reply. If your correspondent had not been in such a hurry he would have seen that in the next issue of the *Medical Temperance Review* for July there is a letter from Dr. Heywood Smith explaining his mistake and apologising for it, with a note from the editor to the effect that he had been misled by the figures which Dr. Heywood Smith had supplied. The means of verifying those figures were not at hand, and there was no antecedent improbability in them. I naturally expected them to have been compiled on the same basis, and if they had been, the remark which your correspondent italicises—that whether they showed the cost per bed or per patient was irrelevant in comparing the expenditure in different hospitals—would have been perfectly correct. Of course, the fact that some represented the amount per bed, and others per patient, makes all the difference as between the different hospitals, and I regret very much that I was thus led to give Middlesex Hospital an undeserved and unenviable notoriety. It was not done with the slightest intention of influencing the charitable public and without any thought of Hospital Sunday.

But it is very good of your correspondent to supply the correct figures, which amply bear out the only inference which I drew from the incorrect ones. For my purpose the hospitals might have been named A, B, C, etc. It was no part of my purpose to attack any one of them. But here we have the hospital which gives least alcohol spending 10s. per bed, and the rest spending an ascending amount up to £2 10s., or five times as much. This your correspondent calls "equable." ("The average cost per bed for alcohol at the various hospitals mentioned is so equable.") If his yearly income varied as much from year to year I doubt not he would describe it very differently. It has varied very much, too, in different years. I happen to have Burdett's book for 1896, which gives the figures for 1894. Just compare them:—

| | Cost of Alcohol per Bed. | | | | | |
|---|---|---|---|---|---|---|
| | 1894. | | | 1899. | | |
| | £ | s. | d. | £ | s. | d. |
| London Hospital | 1 | 15 | 0 | 2 | 5 | 0 |
| Guy's Hospital | 0 | 15 | 0 | 0 | 10 | 0 |
| St. Bartholomew's Hospital | 3 | 5 | 0 | 1 | 15 | 0 |
| St. Thomas's Hospital | 0 | 10 | 0 | 1 | 5 | 0 |
| St. George's Hospital | 3 | 15 | 0 | 2 | 10 | 0 |
| Middlesex Hospital | 2 | 0 | 0 | 1 | 10 | 0 |
| St. Mary's Hospital | 1 | 15 | 0 | 2 | 5 | 0 |
| King's College Hospital | 2 | 15 | 0 | 1 | 10 | 0 |
| University College Hospital | 1 | 5 | 0 | 1 | 5 | 0 |
| Westminster Hospital | 1 | 15 | 0 | 2 | 0 | 0 |
| Charing Cross Hospital | 1 | 15 | 0 | 1 | 10 | 0 |
| Royal Free Hospital | 1 | 0 | 0 | 1 | 0 | 0 |
| Total.. | 22 | 5 | 0 | 19 | 5 | 0 |

The average number of patients in each bed occupied varied only from 12 to 17, the average being 15. But while St. Bartholomew's and Middlesex had (in 1894) the smallest number of patients in each bed (12) it will be seen by the above table that they gave them by no means the least quantity of alcohol. On the other hand St. Thomas's and University College had 17 patients per bed and spent far less. In other words, St. Thomas's spent 10s. for 17 patients and St. Bartholomew's £3 5s. for 12, *i.e.* in 1894. If St. Thomas's had spent as much in proportion for its 17 as Barts for its 12 it would have spent £4 12s.—or more than nine times as much. Possibly the differences between the various hospitals were less last year, as the difference per bed is less; most have improved, St. Bartholomew's having reduced the amount to nearly one half; but four have given more than in 1994.

All this goes to prove my point that alcohol is given without any settled plan. Its administration is still uncertain and depends more on the predilections or fancies of the particular physician or surgeon than on anything else. But if those that give least give enough, it stands to reason that others give much that is unnecessary.

Your correspondent attempts to show that at one or two hospitals which he names the amount each patient gets is very small. Apart from the difficulty that he does not know how many patients did not have any, and therefore that he cannot tell how much the share of each of the rest was, I contend that this does not affect the argument.

Yours truly, J. J. RIDGE,
Hon. Secretary, British Medical Temperance Association.
Carlton House, Enfield.

